# Neonatal Resuscitation in Low Volume Hospital Settings

**DOI:** 10.3390/children9050607

**Published:** 2022-04-25

**Authors:** Ivan Hand

**Affiliations:** 1 Department of Pediatrics, NYC Health + Hospitals/Kings County, Brooklyn, NY 11203, USA; Ivan.Hand@nychhc.org; 2 Department of Pediatrics, SUNY-Downstate College of Medicine, Brooklyn, NY 11203, USA

The vast majority of term newborns will begin breathing and make a successful transition to extrauterine life, whereas a small percentage of infants will require some intervention immediately after birth by a skilled provider [1]. A much higher percentage of preterm infants will require intensive resuscitation efforts at birth for the prevention of death and long-term disability. Perinatal regionalization has been able to improve birth outcomes by bringing high risk pregnancies to high level perinatal centers. The negative consequence of this action is that low volume delivery hospitals have had to care for fewer sick infants and may be less prepared to deal with these newborns. For these reasons, low volume hospitals have higher rates of death and morbidity compared to hospitals with high volume VLBW (very low birthweight) deliveries and tertiary NICUS [2].

In a review by Donohue et al. [3], the authors outline the advantages of telemedicine as a means to train providers in resuscitation techniques as well as assisting in the direct care of the patient. Telemedicine has continued to grow since its inception in the 1990s. With the advent of the COVID-19 pandemic, telemedicine has exploded in the consciousness of providers and the public alike.

From an educational perspective, neonatal resuscitation training is best taught over several sessions as spaced learning [4]. Spaced learning involves multiple training sessions over time as opposed to a single learning event every few years. Academic centers can engage their staff with regular refreshers and “mock codes” but may not be able to provide repeated sessions at a regional affiliate. Telemedicine allows trainees in these low volume regional hospitals to participate in the same training settings as those at the regional center. This has been made even more feasible by the development of patient simulators that can be controlled remotely. Thus, the learners in the affiliate obtain the same experience and benefit from the senior trainer at the academic center.

Telemedicine has also made direct neonatal resuscitation assistance available in remote locations. In a high fidelity simulated neonatal resuscitation, the study participants in a tele-resuscitation group were able to achieve effective ventilation in significantly less time than those in the control group (2 min 42 s vs. 4 min 11 s, *p* < 0.001) [5]. This effectiveness has been documented in real-life situations as well. In a retrospective study assessing the quality of neonatal resuscitation among 92 infants, those receiving telemedicine consults scored significantly better in resuscitation quality than those without consults [6].

Telemedicine allows increased access to neonatal expertise in low volume hospitals and can positively impact the training and care delivered to the newborn. Telemedicine is a cost-effective way to deliver the highest quality of care in settings where a skilled neonatologist may not be immediately available in the birth hospital and allows the remote neonatologist to direct care in these situations, improving neonatal outcomes and supporting the community providers.

## References

[1] Aziz K., Lee H.C., Escobedo M.B., Hoover A.V., Kamath-Rayne B.D., Kapadia V.S., Magid D.J., Niermeyer S., Schmölzer G.M., Szyld E. (2021). Part 5: Neonatal Resuscitation 2020 American Heart Association Guidelines for Cardiopulmonary Resuscitation and Emergency Cardiovascular Care. Pediatrics.

[2] Jensen E.A., Lorch S.A. (2015). Effects of a Birth Hospital’s Neonatal Intensive Care Unit Level and Annual Volume of Very Low-Birth-Weight Infant Deliveries on Morbidity and Mortality. JAMA Pediatr..

[3] Donohue L.T., Hoffman K.R., Marcin J.P. (2019). Use of Telemedicine to Improve Neonatal Resuscitation. Children.

[4] Sawyer T. (2020). Educational Perspectives: Educational Strategies to Improve Outcomes from Neonatal Resuscitation. Neoreviews.

[5] Fang J.L., Carey W.A., Lang T.R., Lohse C.M., Colby C.E. (2014). Real-time video communication improves provider performance in a simulated neonatal resuscitation. Resuscitation.

[6] Fang J.L., Campbell M.S., Weaver A.L., Mara K.C., Schuning V.S., Carey W.A., Colby C.E. (2018). The impact of telemedicine on the quality of newborn resuscitation: A retrospective study. Resuscitation.

